# Effects of different doses of methylprednisolone therapy on acute respiratory distress syndrome: results from animal and clinical studies

**DOI:** 10.1186/s12890-022-02148-y

**Published:** 2022-09-16

**Authors:** Shukun Hong, Chao Jian, Hongye Wang, Xincheng Wang, Luchuan Xing, Lujun Qiao

**Affiliations:** 1 Department of Intensive Care Unit, Shengli Oilfield Central Hospital, Dongying, China; 2 Department of Obstetrics and Gynecology, Dongying Fifth People’s Hospital, Dongying, China; 3 Department of Obstetrics and Gynecology, Shengli Oilfield Central Hospital, Dongying, China

**Keywords:** Glucocorticoids, Methylprednisolone, Acute lung injury, Acute respiratory distress syndrome, Dose, Animal model

## Abstract

**Background:**

The optimal dose of glucocorticoids for acute respiratory distress syndrome (ARDS) is uncertain. This study aimed to evaluate the effects of different doses of methylprednisolone on sepsis-induced acute lung injury (ALI) rats and a cohort of moderate and severe ARDS patients.

**Methods:**

ALI rats, challenged with lipopolysaccharide, were randomly received intraperitoneal injection of normal saline (model group) and different doses of methylprednisolone (0.5, 2, 8 mg/kg, named as low-, moderate- and high-dose group, respectively) for 5 days. The body weight changes of rats, inflammatory factors in bronchoalveolar lavage fluid (BALF), lung wet/dry ratio, histopathological score, and the mRNA expressions of glucocorticoid receptor α (GRα), GRβ and nuclear factor-κB (NF-κB) were measured. Forty moderate and severe ARDS patients were treated with standard of care or plus different doses of methylprednisolone (40, 80, 120 mg/day, named as low-, moderate- and high-dose group, respectively) for 5 days. Clinical outcomes were PaO_2_/FiO_2_ ratio and C-reactive protein (CRP) level at day 5, intubation rate, hospital stay, 28-day mortality, and adverse events rate.

**Results:**

In animal experiment, different doses of methylprednisolone could increase the body weight of rats, and reduce inflammatory factors in BALF and the degree of lung injury compared with model group. The efficacy of methylprednisolone at moderate-dose was better than that at low-dose, but was equivalent to that at high-dose, which was consistent with the differential changes in the mRNA expression of GRα, GRβ and NF-κB. In clinical study, the moderate-dose group was associated with higher PaO_2_/FiO_2_ ratio and lower CRP level. No significant difference in other clinical outcomes among groups was detected.

**Conclusions:**

This study showed that the efficacy of methylprednisolone in ARDS treatment was not always dose-dependent due to the differential regulation of related receptors. The moderate-dose of methylprednisolone may be the potential optimal dose for ARDS treatment, which needs to be further verified by larger clinical trials.

## Introduction

Acute lung injury (ALI)/acute respiratory distress syndrome (ARDS) is the most common acute respiratory failure [1]. It is reported that ARDS accounts for 10.4% of patients in intensive care unit (ICU) and 23.4% of patients with mechanical ventilation [2]. Although great progress has been made in the understanding and management of ARDS in recent decades, its mortality is still as high as 34.9–46.1% [2, 3] which seriously threatens the lives of patients and affects their quality of life. The early pathophysiology of ARDS is characterized by non-cardiogenic pulmonary edema caused by increased permeability of alveolar epithelium and capillary endothelium. This change is closely related to the involvement of multiple inflammatory mechanisms in the lung, including the infiltration of a variety of inflammatory cells dominated by neutrophils and the release of numerous pro-inflammatory mediators, such as tumor necrosis factor-α (TNF-α), interleukin (IL)-1, IL-2, IL-6 and IL-8 [4, 5]. It can be seen that the progression of ARDS may be caused by an uncontrolled inflammatory reaction in the lung. Therefore, anti-inflammatory therapy should be an important part of the management strategy of ARDS.

For a long time, many studies have tried to use glucocorticoids to control the systemic or pulmonary inflammatory responses to achieve the purpose of treating ARDS, but the results are inconsistent [6–9]. Recently, a published meta-analysis of 18 randomized trials demonstrated the role of glucocorticoids in reducing the mortality of ARDS caused by various diseases [10]. However, the specific use of glucocorticoids in the treatment of ARDS is not clear and needs to be further optimized. It is not difficult to find that the dose of glucocorticoids used in previous studies focusing on this topic is variable, which may contribute to the inconsistency of research results. We believe that the clinical efficacy of glucocorticoids in the treatment of ARDS may be closely related to the dose of glucocorticoids, which is worthy of further exploration. In addition, the anti-inflammatory effect of these drugs mainly depends on the glucocorticoid receptor (GR). After hormone binding, GRα can activate anti-inflammatory gene transcriptions by interacting with specific DNA binding domains known as glucocorticoid responsive elements (GREs), and inhibit pro-inflammatory gene transcriptions by interacting with negative GREs [11]. On the contrary, evidence suggests that GRβ may act as a dominant-negative to repress the transcriptional activity of GRα [12]. It is unclear whether different doses of glucocorticoids in the ARDS treatment will regulate the expression of related receptors and thus affect the clinical effects of glucocorticoids.

Therefore, in order to clarify these issues, we first evaluated the effects of different doses of methylprednisolone on sepsis-induced ALI animal models from the level of receptor gene expression. Then, we conducted a small cohort study in patients with moderate and severe ARDS to verify the findings of the animal experiment.

## Materials and methods

### Animals

Thirty SD rats weighing 180–200 g, 6–8 weeks old on arrival, were obtained from the Hubei Research Center of Laboratory Animal (Hubei, China). The animals were kept in a temperature-controlled room with a 12:12-h night-day rhythm and allowed a pelleted diet and water ad libitum.

### Establishment of sepsis-induced ALI model and drug intervention

Rats in the blank control group were injected with 0.5 ml normal saline through the tail vein and received daily intraperitoneal administration of 1 ml normal saline 4 h later for 5 days. Lipopolysaccharide (Sigma, St. Louis, MO) (5 mg/kg) dissolving into normal saline to a final volume of 0.5 ml was infused into the tail vein of the rat to establish the sepsis-induced ALI model. After 4 h of injection, the model rats were randomly divided into 4 groups and received intraperitoneal administration of different agents for 5 days: model group, normal saline; low-dose group, 0.5 mg/kg methylprednisolone (MedChemexpress, Shanghai, China); moderate-dose group, 2 mg/kg methylprednisolone; high-dose group, 8 mg/kg methylprednisolone. There were 6 rats in each group. At the end of treatment, the weight change and survival of rats in each arm were recorded. For further investigation, the rats were sacrificed under sodium pentobarbital anesthesia.

### Measurement of lung wet/dry ratio

The right upper lung tissue was collected, and the water and blood on its surface were removed to obtain the wet weight (W). Then the lung tissue was placed in an oven at 80 ℃ for 72 h and weighed again, recognized as dry weight (D). The W/D ratio was calculated to evaluate the degree of pulmonary edema.

### Measurement of bronchoalveolar lavage fluid (BALF)

After ligating the right main bronchus of the rat, 5 ml of sterile normal saline at 4℃ was injected into the left main bronchus through an infusion tube. Gently turned over the left lung tissue and drew back the liquid for reinjection. Repeating this operation 3 times was counted as one harvest. Three harvests were done to calculate the final volume of BALF.

The collected sample was filtered with single-layer gauze, and 20 ul of BALF was placed on a cell counting plate to count white blood cells under a high-power microscope.

BALF was centrifuged at 300 g for 10 min and the supernatant was removed. The obtained cell mass was fully dispersed, smeared and stained with the Wright-Giemsa method. Neutrophils count was completed under an oil microscope.

The remaining BALF sample was centrifuged at 1000 g at 4 °C for 20 min to remove the cells. The supernatant was collected for IL-6 and TNF-α concentration measurement using ELISA in accordance with the manufacturer’s instructions (Boster Bioengineering Co., Ltd, Wuhan, China).

### Histopathological evaluation

The cleaned lower lobe of the right lung was immersed in 4% formaldehyde solution (Biyuntian Biotech Co., Ltd, Shanghai, China) and fixed for at least 24 h, and then successively subjected to gradient dehydration with ethanol, transparency with xylene, immersion, and embedding in paraffin. The specimens were cut into 4 μm thick segments and stained with hematoxylin and eosin following routine staining procedures. Histopathological evaluation was independently performed under a light microscope by two experienced pathologists blinded for group assignment. According to the scoring criteria of Nishina et al. [13], the following items were evaluated individually: alveolar congestion and hemorrhage, infiltration or aggregation of neutrophils in the airspace or vessel wall, and thickness of alveolar wall/hyaline membrane formation. The scores of each item were added to the final histopathological score.

### PCR for GRα, GRβ and nuclear factor-κB (NF-κB) mRNA expressions

Under sterile conditions, total RNA was extracted from left lung tissues using Trizol reagent (Vazyme Biotech Co., Ltd, Nanjing, China) according to the manufacturer’s instructions. First-strand cDNA was synthesized from the extracted RNA by Hifair® III 1st Strand cDNA Synthesis and SuperMix for qPCR (Yisheng Biotech Co., Ltd, Shanghai, China). The cDNA template was then amplified using MonAmp™ SYBR® Green qPCR Mix (Monad Biotech Co., Ltd, Wuhan, China) by quantitative real-time PCR through the following procedures: pre-denaturation at 95 ℃ for 30 s, followed by 40 cycles, each cycle including denaturation at 95 ℃ for 10 s, annealing at 60 ℃ for 30 s, and extension at 72 ℃ for 20 s. The specific primers were obtained from Sangon Biotech Co., Ltd (Shanghai, China) with the sequences listed in Table 1. The relative expressions of GRα, GRβ and NF-κB were calculated by the ΔΔCT method and normalized to the internal control GAPDH.

**Table 1:** Specific primers for PCR analysis

Gene	Primer sequences	
GAPDH	Forward 5′–3′	GATGCTGGTGCTGAGTATGRCG
	Reverse 5′–3′	GTGGTGCAGGATGCATTGCTCTGA
GRα	Forward 5′–3′	CGTCGGGGACGGATTCTAAG
	Reverse 5′–3′	GCCCAAGTCATTCCCCATCA
GRβ	Forward 5′–3′	TTTTGCGAGCTCGAGTCAGT
	Reverse 5′–3′	ACTGTAGCTCCTCCCCTCAG
NF-κB	Forward 5′–3′	TTCAACATGGCAGACGACGA
	Reverse 5′–3′	GCCATCTGCTGTTGACAGTG

### Patients

From November 2020 to March 2022, patients above 18 years old, who were admitted in the ICU of Shengli Oilfield central hospital (Dongying, China) with moderate and severe ARDS according to the Berlin definition [14], were enrolled. The exclusion criteria were pregnancy, uncontrolled diabetes mellitus and hypertension, patients who had previously been treated with glucocorticoids for various basic diseases, or any contraindications to the use of glucocorticoids, and lack of willingness to participate in the study. All written informed consents signed by the subjects or the authorized representatives were obtained.

### Treatment procedure in clinical investigation

Eligible patients were allocated to the standard of care group or three methylprednisolone groups with different doses. The standard of care group received routine treatment. On this basis, each glucocorticoid group was respectively injected with methylprednisolone 40 mg (low-dose group), 80 mg (moderate-dose group), and 120 mg (high-dose group) intravenously once a day for 5 days.

### Clinical outcomes

The primary outcome was the PaO_2_/FiO_2_ ratio at day 5 following initial treatment. The secondary outcomes were the plasma C-reactive protein (CRP) level at day 5, endotracheal intubation rate, hospital stay, 28-day mortality, and the incidences of adverse events including hyperglycemia, nosocomial infection, and gastrointestinal bleeding.

### Statistical analysis

Statistical analysis was performed using PASW Statistics 18.0 (IBM, New York, NY). Continuous data were expressed as mean ± standard deviation or median (interquartile range) and categorical data were described by number (%). Two-sample t-test was used to compare the body weights of rats before and after treatment. In order to prove the abnormal physiological state of sepsis-induced ALI models, t-test was also used to compare the relevant data of model group and blank control group. One-way analysis of variance was performed to evaluate the differences in experimental outcomes between the model group and the three different concentrations of methylprednisolone treatment groups. For post hoc analysis, the least significance difference procedure would be applied if variances were equal; otherwise, the Games-Howell procedure would be chosen. One-way analysis of variance was also used to compare the continuous variables among four groups of ARDS patients. The χ^2^ test followed by the Bonferroni method was employed to evaluate the comparison of categorical variables. Multivariate Cox regression analysis was applied to identify the potential risk factors that may influence the survival of patients with ARDS. These factors included gender, age > 65 years, comorbidity, PaO_2_/FiO_2_ ratio < 100, Acute Physiology and Chronic Health Evaluation II score > 20, and receiving different doses of methylprednisolone. *P* < 0.05 was considered statistically significant.

## Results

### Weight change and survival

**Fig. 1:**
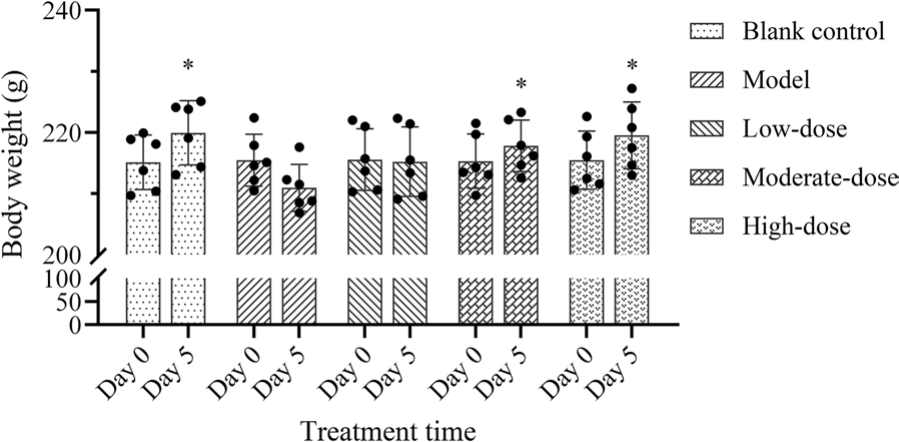
Effects of different doses of methylprednisolone on the body weight of rats. “Day 0”represents the time before treatment, and “Day 5” represents the fifth day of treatment. The height of the bar chart represents the value of the mean, and the black dot at the top of the bar chart represents the individual data from each animal. T-test was used to compare the body weight of rats before and after treatment, and the data between the blank control group and the model group. The comparison between the model group and three different concentrations of methylprednisolone treatment groups was performed by One-way analysis of variance. *P < 0.05 versus model group

The body weights of rats before and after treatment are shown in Fig. 1. After 5 days of treatment, the body weights of rats in the model group and the low-dose group were decreased, while that of the other three groups was increased, but there was no significant difference from that before treatment (*P* > 0.05). Compared with the model group, the body weights of rats in the low-dose group did not change significantly (*P* > 0.05), while that in the moderate- and high-dose groups increased significantly (*P* < 0.05). However, there was no significant difference between the latter two groups (*P* > 0.05). At the end of treatment, one animal died in the model group, the other rats all survived.

### W/D ratio of lung

**Fig. 2:**
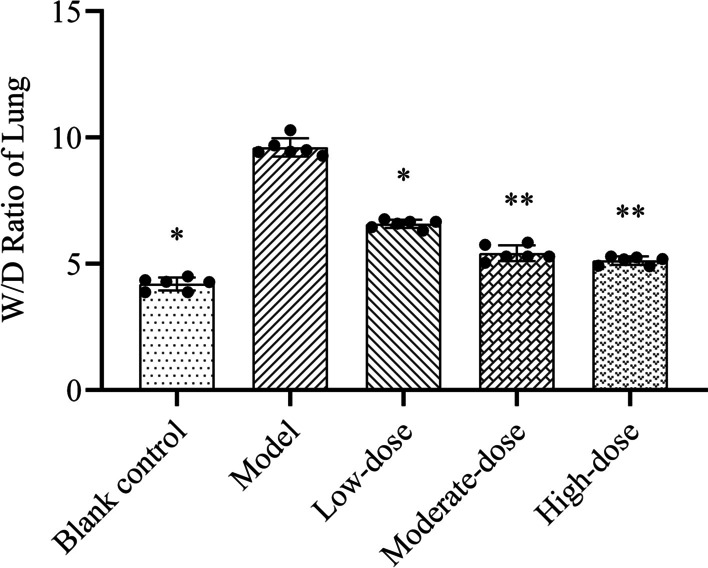
Effects of different doses of methylprednisolone on the lung wet/dry ratio in rats. The height of the bar chart represents the value of the mean, and the black dot at the top of the bar chart represents the individual data from each animal. T-test was used to compare the data between the blank control group and the model group. The comparison between the model group and three different concentrations of methylprednisolone treatment groups was performed by One-way analysis of variance. *P < 0.01 versus model group; **P < 0.01 versus low-dose group

The W/D ratio of rat lung in the model group was significantly higher than that in the blank control group (*P* < 0.05). The ratio in each methylprednisolone treatment group was significantly lower than that in the model group (*P* < 0.05). Compared to the low-dose group, the ratios in the moderate- and high-dose groups were also significantly reduced (*P* < 0.05). However, no significant difference in the W/D ratio was observed between the moderate- and high-dose groups (*P* > 0.05) (Fig. 2).

### White blood cell and neutrophil counts in BALF

**Fig. 3:**
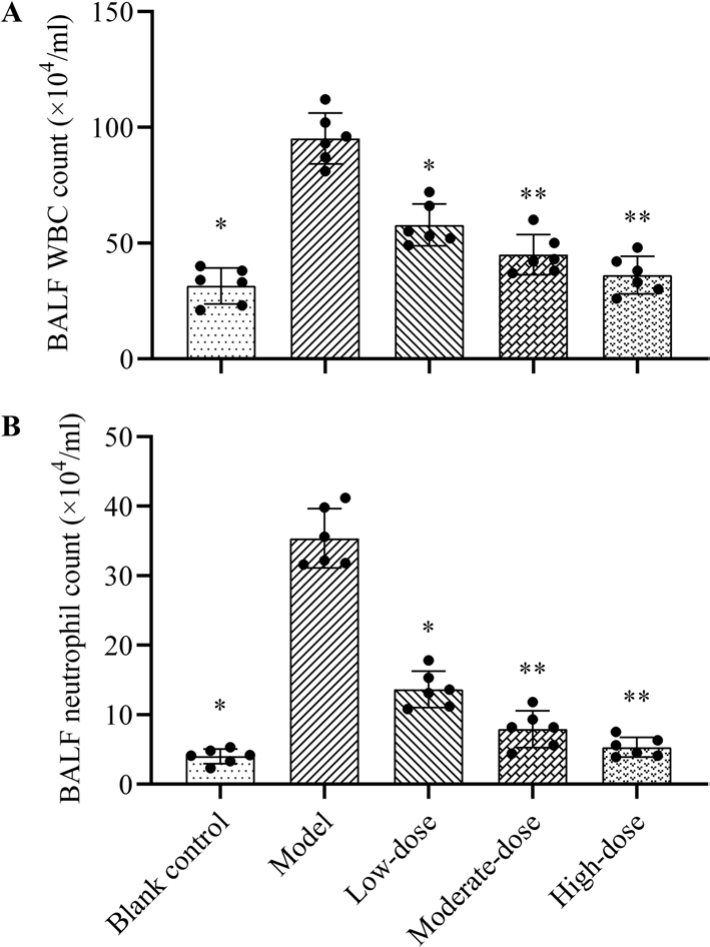
Effects of different doses of methylprednisolone on the WBC and neutrophil counts in BALF of rats. **A** WBC count; **B** neutrophil count. The height of the bar chart represents the value of the mean, and the black dot at the top of the bar chart represents the individual data from each animal. T-test was used to compare the data between the blank control group and the model group. The comparison between the model group and three different concentrations of methylprednisolone treatment groups was performed by One-way analysis of variance. *P < 0.01 versus model group; **P < 0.05 versus low-dose group

In this experiment, the recovery rate of the BALF was higher than 80%. As shown in Fig. 3, the white blood cell and neutrophil counts of BALF in the model group were significantly raised than those in the blank control group (*P* < 0.05). After methylprednisolone treatments, the counts of white blood cells and neutrophils in BALF were significantly decreased than those in the model group (*P* < 0.05). Compared with low-dose methylprednisolone, moderate- and high-dose glucocorticoid treatments could significantly reduce the white blood cell and neutrophil counts in BALF (*P* < 0.05), but the difference between the latter two groups was not statistically significant (*P* > 0.05).

### Concentrations of IL-6 and TNF-α in BALF

**Fig. 4:**
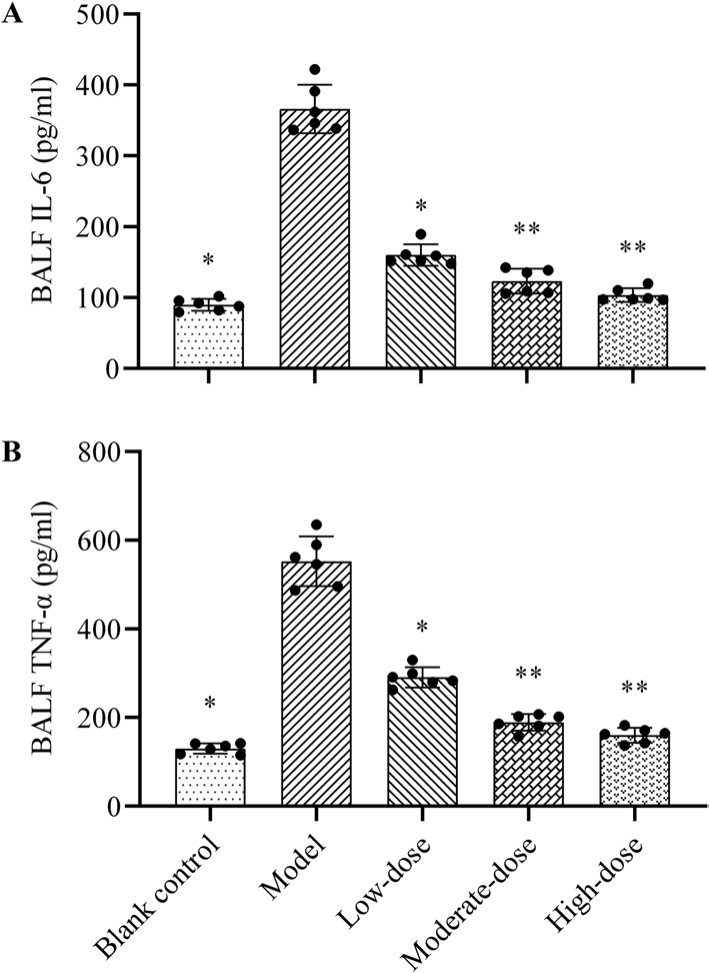
Effects of different doses of methylprednisolone on the concentrations of IL-6 and TNF-α in BALF of rats. **A** IL-6 concentration; **B** TNF-α concentration. The height of the bar chart represents the value of the mean, and the black dot at the top of the bar chart represents the individual data from each animal. T-test was used to compare the data between the blank control group and the model group. The comparison between the model group and three different concentrations of methylprednisolone treatment groups was performed by One-way analysis of variance. *P < 0.01 versus model group; **P < 0.05 versus low-dose group

As illustrated in Fig. 4, the concentrations of IL-6 and TNF-α in BALF in the model group were significantly elevated than those in the blank control group (*P* < 0.05). Different concentrations of methylprednisolone could significantly reduce the levels of IL-6 and TNF-α in BALF compared to the model group (*P* < 0.05), whereas the decrease was not hormone dose-dependent. In contrast to low-dose, both moderate- and high-dose glucocorticoids could significantly reduce the BALF concentrations of IL-6 and TNF-α (*P* < 0.05), but there was no significant difference in this effect between these two groups (*P* > 0.05).

### Histopathological evaluation

**Fig. 5:**
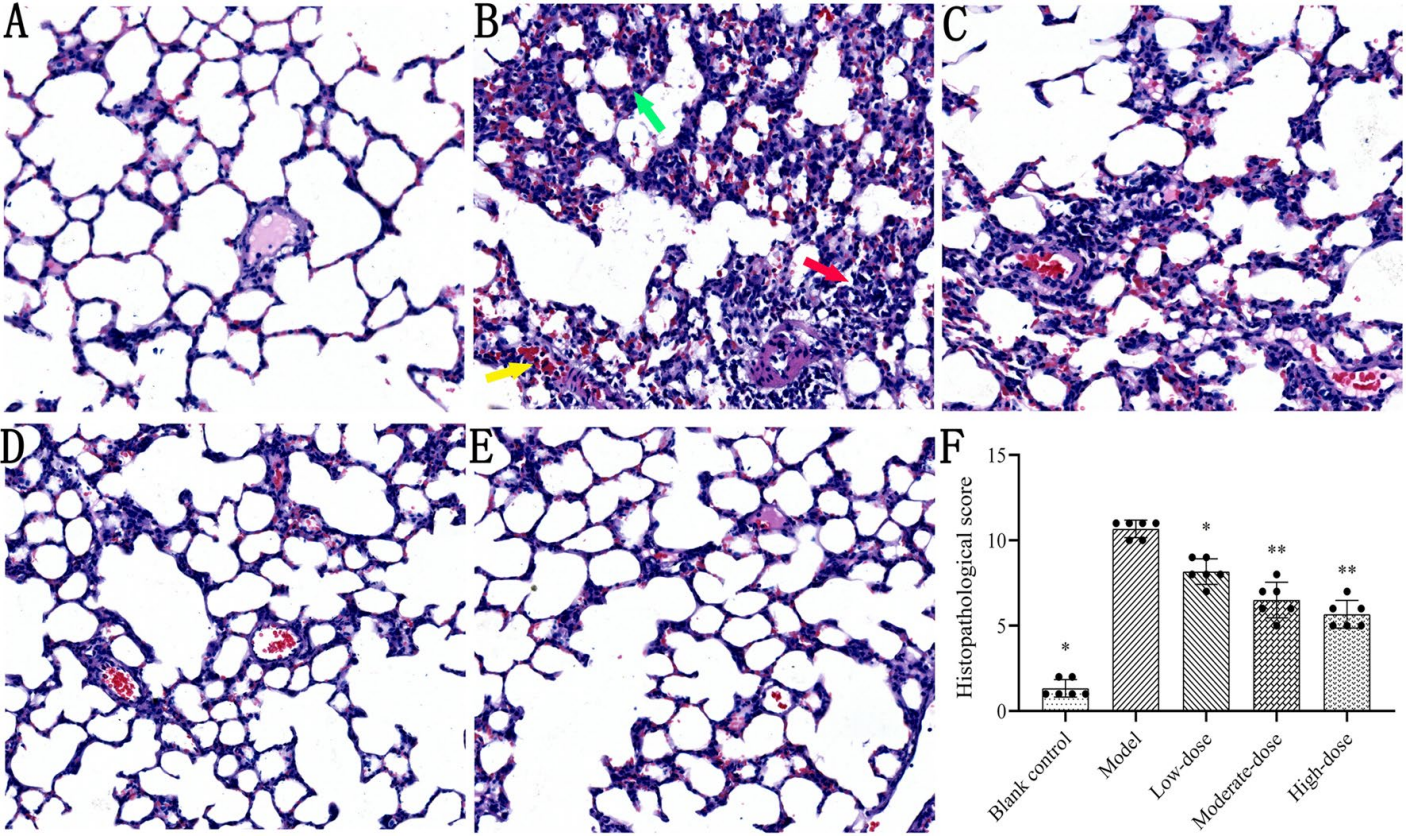
Effects of different doses of methylprednisolone on histopathological change of lung tissue in rats. Hematoxylin-and-eosin-stained sections of lung tissue were observed under a light microscope (original magnification × 100). Representative image of each group was revealed. **A** blank control group; **B** model group, yellow arrow indicates the alveolar hemorrhage, red arrow indicates the infiltration or aggregation of neutrophils in the airspace, and green arrow indicates the thickening of alveolar wall; **C** low-dose group; **D** moderate-dose group; **E** high-dose group. **F** histopathological scores of lung tissues in each group. The height of the bar chart represents the value of the mean, and the black dot at the top of the bar chart represents the individual data from each animal. T-test was used to compare the data between the blank control group and the model group. The comparison between the model group and three different concentrations of methylprednisolone treatment groups was performed by One-way analysis of variance. *P < 0.01 versus model group; **P < 0.01 versus low-dose group

Figure 5 shows the typical histopathological manifestations of lung tissues in each group. In the blank control group, the alveolar structure was intact, no exudation and bleeding in the alveolar cavity were observed, the alveolar septum was not widened, and there was a small amount of leukocyte infiltration in the lung stroma (Fig. 5A). In contrast, macroscopic bleeding and exudation in the gross specimens of the lung were observed in the model group. The histology of lung tissue was significantly changed, including the destruction of alveolar structure, alveolar collapse, diffuse alveolar, and interstitial hemorrhage, interstitial exudation and thickening, formation of hyaline membranes, and infiltration of inflammatory cells in the interstitial space (Fig. 5B). After treatment with different concentrations of methylprednisolone, the lung tissue injuries of rats were significantly reduced in varying degrees based on histopathological scores (*P* < 0.05) (Fig. 5C–F). Nevertheless, the difference in histopathological scores between moderate- and high-dose groups was not significant (*P* > 0.05) (Fig. 5F).

### GRα, GRβ and NF-κB mRNA expression

**Fig. 6:**
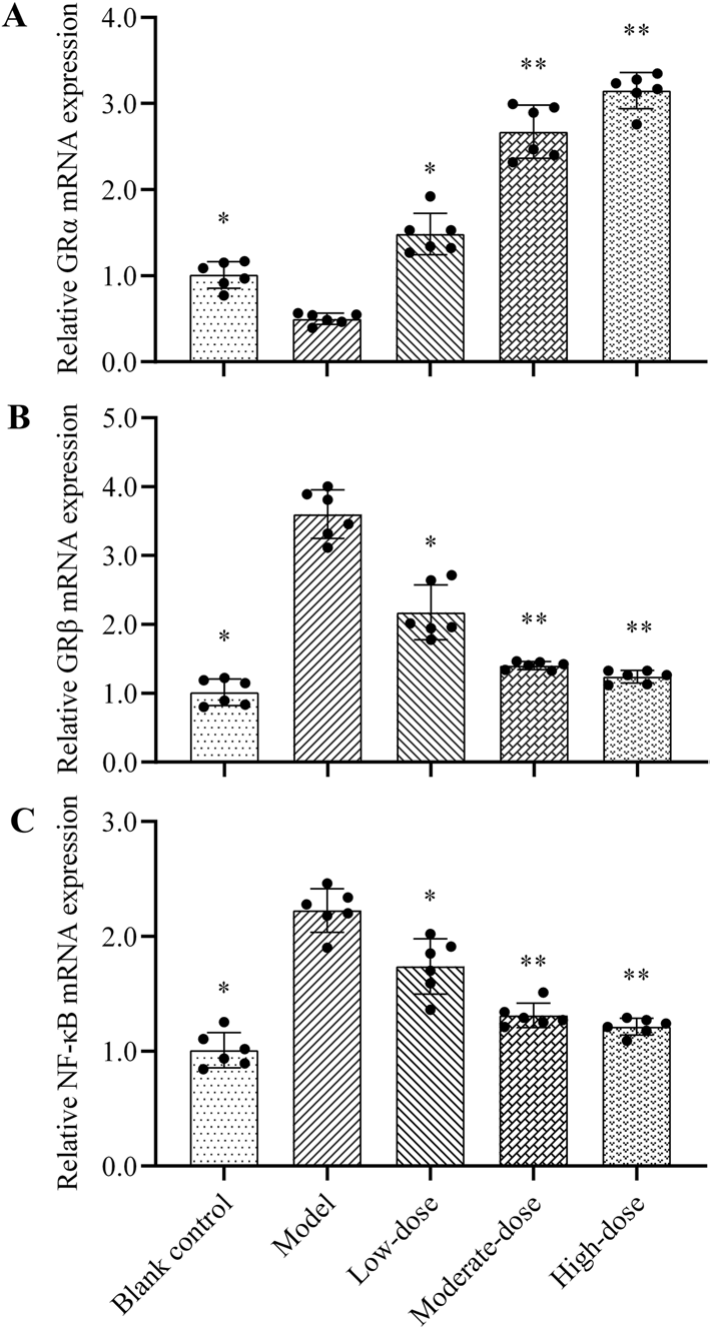
Effects of different doses of methylprednisolone on mRNA expression of GRα, GRβ and NF-κB in lung tissue of rats. **A** GRα mRNA expression; **B** GRβ mRNA expression; **C** NF-κB mRNA expression. The height of the bar chart represents the value of the mean, and the black dot at the top of the bar chart represents the individual data from each animal. T-test was used to compare the data between the blank control group and the model group. The comparison between the model group and three different concentrations of methylprednisolone treatment groups was performed by One-way analysis of variance. *P < 0.05 versus model group; **P < 0.05 versus low-dose group

The GRα, GRβ and NF-κB mRNA expressions of each group after experimental treatment were displayed in Fig. 6. Compared with the blank control group, the expression of GRα mRNA in the model group was reduced significantly (*P* < 0.05). With the increase of hormone treatment dose, the expression of GRα mRNA was increased accordingly. However, there was no significant difference in GRα mRNA expression between the moderate- and high-dose glucocorticoid treatments (*P* > 0.05). On the contrary, the GRβ and NF-κB mRNA expressions were significantly higher in the model group than in the blank control group (*P* < 0.05), and decreased gradually with the increase of hormone dose. Likewise, there was no significant difference between the moderate- and high-dose groups in the mRNA expression of GRβ and NF-κB (*P* > 0.05).

### Results of clinical investigation

A small cohort of 40 patients with moderate and severe ARDS was enrolled in this clinical study, with 10 in each group. The baseline demographic and clinical characteristics of patients were shown in Table 2. There was no significant variation among the four groups based on these baseline data. After treatment, the PaO_2_/FiO_2_ ratio at day 5 in the moderate-dose group was significantly higher than that in the standard of care group (262.49 vs. 160.23, *P* < 0.05) and the low-dose group (262.49 vs. 156.63, *P* < 0.05), but there was no significant difference from that in the high-dose group (262.49 vs. 222.49, *P* > 0.05) (Table 3). The CRP level at day 5 in the moderate-dose group was significantly lower than that in the control group (51.06 vs. 138.96, *P* < 0.05), but there was no significant difference from that in the low-dose group (51.06 vs. 118.92, *P* > 0.05) or the high-dose group (51.06 vs. 72.27, *P* > 0.05) (Table 3). No significant difference in other clinical outcomes among the groups was observed (Table 3). However, there was a trend that the intubation rate, 28-day mortality, and the incidences of adverse events decreased most in the moderate-dose group. The multivariate Cox regression analysis found no independent risk factors that could affect the survival of patients with ARDS.

**Table 2:** Baseline demographic and clinical characteristics of patients at baseline

	Total patients (n = 40)	Standard of care (n = 10)	Lose-dose MP (n = 10)	Moderate-dose MP (n = 10)	High-dose MP (n = 10)	*P*-value
Age (years)	64.35 ± 19.18	66.4 ± 19.78	66.1 ± 12.64	56.7 ± 24.15	68.2 ± 19.26	0.550
Sex (male, %)	31 (77.5%)	7 (70%)	8 (80%)	8 (80%)	8 (80%)	0.930
*Comorbidities*						
Hypertension, n (%)	20 (50%)	5 (50%)	3 (30%)	6 (60%)	6 (60%)	0.494
Diabetes, n (%)	12 (30%)	5 (50%)	2 (20%)	4 (40%)	1 (10%)	0.190
Cardiac disease, n (%)	9 (22.5%)	4 (40%)	0 (0%)	4 (40%)	1 (10%)	0.063
Respiratory disease, n (%)	2 (5%)	1 (10%)	0 (0%)	1 (10%)	0 (0%)	0.526
Renal disease, n (%)	6 (15%)	4 (40%)	1 (10%)	1 (10%)	0 (0%)	0.070
Brain disease, n (%)	12 (30%)	4 (40%)	2 (20%)	3 (30%)	3 (30%)	0.813
*Etiology of ARDS*						
Severe pneumonia	22 (55%)	6 (60%)	5 (50%)	5 (50%)	6 (60%)	0.939
Trauma	13 (32.5%)	2 (20%)	4 (40%)	4 (40%)	3 (30%)	0.740
Sepsis	5 (12.5%)	2 (20%)	1 (10%)	1 (10%)	1 (10%)	0.877
PaO_2_/FiO_2_ ratio	118.26 ± 33.85	110.43 ± 38.63	129.75 ± 35.14	110.58 ± 28.18	122.27 ± 33.85	0.520
White blood cell count	13.42 ± 7.84	13.90 ± 9.13	9.71 ± 4.72	15.12 ± 7.68	14.71 ± 9.22	0.613
C-reactive protein	126.68 ± 98.08	183.96 ± 116.83	113.15 ± 89.49	103.94 ± 59.71	104.31 ± 106.4	0.200
Procalcitonin, median (IQR)	8.72 ± 25.29	5.66 (0.49–16.20)	2.74 (1.16–6.10)	1.20 (0.28–7.20)	1.31 (0.85–5.92)	0.392
APACHE II score	29.91 ± 8.95	31.22 ± 13.19	30.14 ± 10.11	28.63 ± 7.96	29. 56 ± 3.25	0.950

Data are presented as mean ± standard deviation or n (%), unless otherwise stated

*MP* methylprednisolone, *ARDS* acute respiratory distress syndrome, *IQR* interquartile range, *APACHE* Acute Physiology and Chronic Health Evaluation

**Table 3:** Secondary Study outcomes in each group in the clinical studyinvestigation

Outcomes	Standard of care (n = 10)	Lose-dose MP (n = 10)	Moderate-dose MP (n = 10)	High-dose MP (n = 10)
PaO_2_/FiO_2_ ratio at day 5	160.23 ± 66.41	156.63 ± 71.01	262.49 ± 68.55*^△^	222.49 ± 94.87
C-reactive protein at day 5	138.96 ± 65.67	118.92 ± 91.83	51.06 ± 51.97*	72.27 ± 93.40
Intubation, n (%)	4 (57%)	2 (50%)	2 (33%)	4 (66%)
Hospital stay (days)	49.00 ± 17.64	35.33 ± 17.34	29.86 ± 20.95	27.20 ± 17.54
28-day mortality, n (%)	4 (40%)	3 (30%)	1 (10%)	5 (50%)
*Adverse events*				
Hyperglycemia, n (%)	9 (90%)	7 (70%)	6 (60%)	8 (80%)
Nosocomial infection, n (%)	6 (60%)	6 (60%)	4 (40%)	7 (70%)
Gastrointestinal bleeding, n (%)	0 (0%)	0 (0%)	0 (0%)	1 (10%)

Data are presented as mean ± standard deviation or n (%)

*MP* methylprednisolone

*P < 0.05 versus standard of care; ^△^*P* < 0.05 versus low-dose MP

## Discussion

Our animal experiment demonstrated that the increase of inflammatory cytokines and the occurrence of pulmonary edema were related to the decreased expression of GRα mRNA and the increased expression of GRβ and NF-κB mRNAs in sepsis-induced ALI, and that the early treatment of methylprednisolone could significantly increase the body weight of rats, lower the level of inflammatory cytokines, and alleviate the degree of lung injury, and that the efficacy of methylprednisolone at moderate-dose was better than that at low-dose, but was equivalent to that at high-dose, which was consistent with the differentiated changes in the mRNA expression of the three receptors. Our clinical study also demonstrated that the moderate-dose of methylprednisolone was associated with a better improvement in patients with moderate and severe ARDS. These findings suggest that the anti-inflammatory effect of methylprednisolone is not always dose-dependent, and high-dose glucocorticoids may have no advantages in the treatment of ARDS.

Sepsis is the leading cause of ARDS and accounts for 30% of the etiology of ARDS [15]. When sepsis occurs, the activated immune system mediates the host to produce a lot of inflammatory factors, which is very important for pathogen clearance [16]. However, high levels of inflammatory cytokines can damage the endothelium or epithelium of the lung, leading to ARDS [17]. In our study, the animal model of sepsis-induced ALI was established by injecting lipopolysaccharide into the tail vein of rats. Compared with the blank control group, the inflammatory factors including white blood cells, neutrophils, IL-6 and TNF-α in BALF in the model group were significantly increased, demonstrating the existence of inflammatory response in the lung after endotoxin infusion. The elevated W/D ratio of the lung implied the increase of extravascular lung water content and alteration of the alveolar-capillary barrier [18]. In terms of histopathology, the evidence of lung tissue injury consisted of the collapse of alveolar structure, hemorrhage in the alveolar and interstitial space, interstitial thickening, formation of hyaline membranes, and accumulation of inflammatory cells in the interstitial space. According to the above results, the animal model of sepsis-induced ALI in this study could be considered to be successfully established [19].

There exists a large body of evidence demonstrating the vital role of transcription factor NF-κB activation in sepsis [20]. Our study showed that the mRNA expression of NF-κB was increased significantly in the model group, suggesting that the transcription pathway of pro-inflammatory factors induced by NF-κB may contribute to the occurrence and development of sepsis-induced ALI. Given the correlation between the pathogenesis of the disease and excessive inflammatory response, inhibiting or reducing the production of inflammatory mediators can be regarded as the focus of treatment.

Previous studies have confirmed that glucocorticoids can simultaneously act on two transcription pathways induced respectively by GRα and NF-κB to exert their anti-inflammatory effects. Briefly, on the one hand, glucocorticoid binding triggers a conformational change in the GRα that provokes its dissociation from chaperone proteins, exposing the nuclear localization signal of GRα and allowing it to translocate to the nucleus. Once in the nucleus, GRα can activate anti-inflammatory gene transcriptions by interacting as a homodimer with GREs located in the promoter regions of target genes, and inhibit pro-inflammatory gene transcriptions by interacting with negative GREs [11, 21]. On the other hand, the active subunit p65 of NF-κB is directly bound by the activated GRα, blocking the binding of NF-κB to the target site on DNA. At the same time, GRα can increase the transcription level of NF-κB inhibitor (IκB), making NF-κB recombine by IκB and turn into an inactive state, failing NF-κB to activate pro-inflammatory gene transcription [12].

More than a decade ago, Meduri et al. [22] conducted a randomized trial and found that the use of low-dose (1 mg/kg/day) methylprednisolone in patients with early severe ARDS could reduce serous C-reactive protein levels, and improve pulmonary and extrapulmonary organ function. After that, Wang et al. [23] investigated in an animal experiment of sepsis-induced ALI that low-dose dexamethasone could reduce inflammatory factors in BALF fluid and upregulate GR mRNA expression in lung tissue. However, the specific subtype of GR in that experiment was not described. In the present study, we found that low-dose (0.5 mg/kg) methylprednisolone could significantly increase the body weight of rats, decrease the release of inflammatory cytokines, and attenuate the histopathological damage. These outcomes were closely associated with increased GRα and decreased NF-κB mRNA expression.

Another experiment [24], in which the ALI model was established by administrating lipopolysaccharide intratracheally, reported that methylprednisolone at a dose of 2 mg/kg attenuated lung mechanical changes, cytokine levels in BALF, and the mRNA expression of inflammatory indexes in lung tissue. Our animal study had similar findings and further showed that the anti-inflammatory effect of moderate-dose (2 mg/kg) methylprednisolone was stronger than that of low-dose (0.5 mg/kg). However, it was interesting to find that this anti-inflammatory effect did not change in a dose-dependent manner with the increase of hormone dose. There was no significant difference in body weight, inflammatory factor level, and degree of lung injury between the high-dose (8 mg/kg) group and the moderate-dose (2 mg/kg) group. Although the expression of related genes in the moderate-dose group was different from that in the low-dose group, it was equivalent to that in the high-dose group. This dose-independent effect of methylprednisolone was also recorded in the animal experiment of Song et al. [25]. The researchers treated the rat model of smoke inhalation-induced ALI with different doses (0.4, 4, 40 mg/kg) of methylprednisolone for 3 and 7 days. The results showed that compared with the other two doses, the dose of 4 mg/kg was related to reduced pro-inflammatory cytokine production and neutrophil infiltration into alveoli, as well as improved survival rate. These phenomena suggest that the anti-inflammatory efficacy of methylprednisolone in the treatment of ARDS is not always dose-dependent. Therefore, we speculate that there may be an optimal dose, which can optimize the expression of receptors mediating anti-inflammatory and pro-inflammatory pathways, and exert the maximum anti-inflammatory effectiveness of methylprednisolone. Hormone treatment with a higher dose above this threshold may no longer increase its anti-inflammatory property, and may even produce harmful effects.

Several clinical studies reported that high-dose glucocorticoid therapy could not improve the mortality of patients with ARDS [26–29]. An animal study [30], comparing the effects of different doses (3, 30, 180 mg/kg) of methylprednisolone on sepsis-induced ALI rats, found that continuous administration of 180 mg/kg methylprednisolone within 1–2 weeks aggravated lung injury, which was characterized by the increased lung injury score, the W/D ratio and the total number of cells in BALF. Furthermore, a retrospective study by Kido et al. discovered that there was increased mortality in ARDS patients receiving high-dose glucocorticoid treatment [31].

These findings all point to the fact that high-dose methylprednisolone is ineffective in the treatment of ALI. However, few studies have explained the above phenomena at the level of receptors mediating inflammatory pathways. Based on the outcomes of the present study, the following possible reasons can be considered. Firstly, there was no significant difference in GRα mRNA level between moderate- and high-dose groups, meaning that further increasing the dosage of the hormone could not increase GRα gene expression, thus the anti-inflammatory effect induced by GRα could not be enhanced. Secondly, the expression of NF-κB mRNA in the high-dose group was not significantly lower than that in the moderate-dose group, indicating that the stimulation of high-dose hormone could not weaken the pro-inflammatory effect mediated by NF-κB. Thirdly, the efficacy of glucocorticoids may be impaired by the dominant-negative activity of GRβ, which is traditionally recognized as an endogenous antagonist of GRα.

In contrast to GRα, GRβ cannot bind glucocorticoids due to its altered ligand-binding domain [11]. Despite this, it can inhibit the GRα-induced transcriptional activity in multiple cell types [32]. The mechanism of its negative effect on GRα mainly includes heterodimerization with GRα and competition for GRE binding [33]. In addition, GRβ has intrinsic, GRα-independent transcriptional activity that can directly activate inflammatory pathways [33]. Previous reports on the physiological function of GRβ mainly focused on the relationship with glucocorticoid resistance [21, 34–37]. The present study found an increased expression of GRβ mRNA in the model group, which may be the result of the stimulation of elevated pro-inflammatory cytokines [38]. At the same time, this may indicate the existence of endogenous glucocorticoid resistance. Similarly, the down-regulation of GRβ expression in low- and moderate-dose groups might be related to the decrease of those cytokines. Yu et al. [39] found that high-dose or long-term glucocorticoid treatments could up-regulate the expression of GRβ and cause glucocorticoid resistance, which limits the effect of glucocorticoids. However, in our study, the GRβ expression in the high-dose group was neither increased nor further decreased compared with that in the moderate-dose group. Combined with the findings of Yu et al., we speculate that this may be a turning point in the change of GRβ expression, which may mean that the exogenous glucocorticoid resistance begins to appear. Furthermore, it is worth noting that GRβ binds GRE-containing DNA with a greater capacity than GRα in the absence of glucocorticoids [32], suggesting that the effect of GRα may be easily influenced by fewer GRβ changes. Taken together, these properties of GRβ may contribute to the ineffective effects of high-dose methylprednisolone observed in the present study.

Based on the above analyses, we infer that the optimal dose of glucocorticoid for the treatment of ARDS may be moderate-dose. This inference has been preliminarily verified in our small cohort study, which is reflected in that the moderate-dose of methylprednisolone could significantly increase the PaO_2_/FiO_2_ ratio and reduce the CRP level in patients with moderate and severe ARDS compared with the other two doses. Moreover, moderate-dose of methylprednisolone may have potential advantages in reducing mortality, intubation rate, and adverse events, although these outcomes did not differ significantly between groups, which could not rule out the possibility of false-negative results due to the small sample size. Besides, it is reported that the saturation of genomic expression modulation will be achieved at the moderate glucocorticoid dose levels (80–100 mg) [40]. This is consistent with the findings of our clinical study.

There are some limitations in the present study, which deserve discussion. First, the glucocorticoid used in this study is methylprednisolone, whether other glucocorticoids will produce the same results is unknown. Some studies suggest that compared with other glucocorticoids, methylprednisolone will revert the largest number of the gene perturbed by virus infection [41], and has stronger non-genomic effects on epithelial cells and higher penetration in lung tissue [40]. Thus, it may play a better clinical effect in lung diseases. Unfortunately, the present study did not conduct investigations on the kinetics of methylprednisolone treatment, which may be an important factor for treatment effect. Second, the duration of drug intervention designed in this study is short-term (5 days), which is in line with the habit of clinicians using methylprednisolone to treat patients with lung diseases. Whether longer courses or higher doses of glucocorticoid application will have different results still needs to be further studied. For example, the animal study [30] mentioned above reported that administration of methylprednisolone at a dose of 180 mg/kg for 14 days could aggravate lung injury of ALI rats, but the study did not carry out molecular biology experiments. If predicted according to the results of our study, the expression of NF-κB and GRβ in that study would be relatively increased, leading to glucocorticoid resistance and decreased efficacy or inefficacy. We note that the dose range of methylprednisolone used in rats may be relatively narrow from the level of animal experiments. However, if this range is applied to the real world, it may be easier for research institutions to accept and carry out corresponding clinical investigations. Third, we realize that genomic effects are achieved through protein expression. Regrettably, at the molecular level, our study only detected the mRNA expression of receptors, while did not measure the expression of related proteins. This is because the antibody reagent for detecting GRβ protein is very rare and we cannot obtain it. As a result, experiments for protein measurement were canceled, which may be a defect of our study. Besides, the sample size of rats and participants in this study is relatively small, and the gender composition in clinical research is unbalanced. These possible biases may partly affect the research results that need to be interpreted with caution. Despite the above limitations, however, our study can be considered as a preliminary investigation to explore the optimal dose of glucocorticoids in the treatment of ARDS. The findings of this study may provide some references for clinicians to choose the dose of methylprednisolone in the ARDS treatment in the future. Given the expression changes of GRβ observed in this study, the possible drug resistance of glucocorticoids in ARDS treatment may be worth further exploration.

## Conclusions

Our animal study showed that different doses of methylprednisolone had a therapeutic effect on sepsis-induced ALI, while this effect was not always dose-dependent. The efficacy of moderate-dose methylprednisolone was better than that of low-dose and was equivalent to that of high-dose. This change in efficacy was associated with the increase of GRα mRNA expression and the reduction of GRβ and NF-κB mRNA expressions. Our clinical study demonstrated that methylprednisolone at a moderate-dose was associated with a better improvement in patients with moderate and severe ARDS. Based on the findings of our study, we believe that the moderate-dose of methylprednisolone may be the potential optimal dose for the treatment of ARDS, which needs to be further verified by larger clinical trials.

## Data Availability

The datasets analyzed during the present study are available from the corresponding author on reasonable request.

## Abbreviations

ALI: Acute lung injury
ARDS: Acute respiratory distress syndrome
BALF: Bronchoalveolar lavage fluid
CRP: C-reactive protein
D: Dry weight
GR: Glucocorticoid receptor
GREs: Glucocorticoid responsive elements
ICU: Intensive care unit
IL: Interleukin
NF-κB: Nuclear factor-κB
IκB: NF-κB inhibitor
TNF-α: Tumor necrosis factor-α
W: Wet weight
